# Covid-19 vaccination acceptance and hesitancy among the Turkish adult population

**DOI:** 10.3205/dgkh000427

**Published:** 2023-01-17

**Authors:** Sükran Köse, Aliye Mandiracioglu, Yusuf Özbel, Seheray Zeyrek, Didem Dereli Akdeniz, Hossein Samadi Kafil

**Affiliations:** 1 Department of Infectious Diseases and Clinical Microbiology, Dokuz Eylül Üniversitesi, İzmir, Turkey; 2 Ege University Faculty of Medicine, Department of Parasitology, Bornova, İzmir, Turkey; 3 Ege University Faculty of Medicine, Department of Public Health, Bornova, İzmir, Turkey; 4 Department of Endochronology, Faculty of Medicine, Bakirçay University, Izmir, Turkey; 5 Drug Applied Research Center, Faculty of Medicine, Tabriz University of Medical Sciences, Tabriz, Iran

**Keywords:** Covid-19, vaccine, risk, protectionimmunization

## Abstract

**Objective:**

The aim of this study was to determine the acceptance of Covid-19 vaccine among the Turkish adult population.

**Methods:**

A total of 2023 persons participated in this cross-sectional study between October 2020 and January 2021. The questionnaire, which was delivered via social media, was filled out by the participants over “Google Forms”.

**Results:**

Questionnaire results showed that 68.7% of the participants might agree to vaccinated against COVID-19. According to univariate analysis, the age group of 50–59, urban residents, healthcare workers, non-smokers, and those with chronic diseases, those who were vaccinated against influenza, pneumonia, and tetanus were all willing to be vaccinated against COVID-19.

**Conclusions:**

It is very important to determine a community’s willingness to be vaccinated against COVID-19 so that interventions can be made to solve related problems. Risk of exposure and importance of Prevention play a critical role in vaccination acceptance.

## Zusammenfassung

**Zielsetzung:**

Ziel der Studie war es, die Akzeptanz der Covid-19 Impfung in der türkischen Erwachsenenbevölkerung zu ermitteln.

**Methode:**

An der Querschnittsstudie nahmen zwischen Oktober 2020 und Januar 2021 2023 Personen teil. Der Fragebogen, der über soziale Medien übermittelt wurde, wurde von den Teilnehmern über „Google Forms“ ausgefüllt.

**Ergebnisse:**

68,7% der Teilnehmer stimmen der Impfung gegen COVID-19 zu. Die univariate Analyse ergab, dass die Altersgruppe der 50- bis 59-Jährigen, Stadtbewohner, Beschäftigte im Gesundheitswesen, Nichtraucher und chronisch Kranke sowie diejenigen, die gegen Influenza, Lungenentzündung und Tetanus geimpft waren, alle bereit waren, sich gegen COVID-19 impfen zu lassen.

**Schlussfolgerung:**

Es ist wichtig, die Bereitschaft einer Gemeinschaft zu ermitteln, sich gegen COVID-19 impfen zu lassen, damit Maßnahmen zur Lösung der damit verbundenen Probleme ergriffen werden können. Das Expositionsrisiko und die Erkennung von Gefahren spielen eine entscheidende Rolle für die Akzeptanz der Impfung.

## Introduction

The spread of COVID-19 has caused a massive human and economic crisis around the world. Controlling the transmission of SARS-CoV-2 is an urgent public-health priority [1]. Therefore, there was an unprecedented global effort to develop a vaccine against this disease. Policymakers struggling to implement traditional preventive measures in controlling COVID-19 disease have been preparing for the next challenge: vaccination acceptance in the community [2]. COVID-19 vaccination hesitation arises with doubts about its safety, especially since the vaccine is new. The WHO recognized it as one of the ten largest global health risks in 2019. Vaccination hesitation and disinformation in many countries pose great challenges in ensuring vaccine coverage and community immunity. A society’s readiness, acceptance and desire for vaccination are important in organizing vaccination services. In order to control the pandemic with the vaccine, a critical proportion of the society must be reached and immunized [3], [4].

The success of the vaccination program depends on the rate of vaccination in the community. It is very important to develop effective vaccination policies to successfully implement wide-spread vaccination. Health behaviors, including vaccination acceptance, are explained by the Health Belief Model, the Theory of Planned Behavior, and Protection Motivation Theories. Structures such as threat, coping, action, self-efficacy, perceived benefits and barriers, subjective norms, perceived behavior control and attitudes explained by these theories were associated with vaccination during the H1N1 influenza pandemic. On the other hand, the current pandemic involves a new disease, and how the new COVID vaccine is depicted in the media affects its acceptance in the community [5]. The degree of trust individuals place in policy makers and public health authorities has been associated with an individual’s willingness to participate in public health measures such as vaccinations [6]. The Health Belief Model (HBM) has been one of the most widely used theories in understanding health and disease behavior. HBM consists of several main structures: perceived sensitivity, perceived seriousness, perceived benefits, perceived barriers, self-efficacy to act, and action. Perceived sensitivity refers to beliefs about vulnerability to infection, while perceived Seriousness refers to beliefs about the negative effects of contracting infection. Perceived benefits related to vaccination are defined as an individual’s beliefs about getting vaccinated, and perceived barriers are defined as the belief that vaccination is restricted due to psychosocial, physical, or financial factors [7].

Vaccine hesitancy is defined as delay in accepting vaccination or rejecting vaccination despite the availability of vaccination services. Vaccine hesitancy is a complex phenomenon, which may vary depending on time, location and vaccine. It may be affected by factors such as indifference, convenience, and trust [8]. Vaccine hesitation and disinformation in many countries present enormous difficulties in ensuring inclusion and community immunity. Coping with these situations and protective behaviors are important in pandemic management. Research into the acceptance of the Covid-19 vaccine has gained importance in developing future strategies [9]. There are many factors that affect the vaccination decision. It is important to identify these in vaccination services and to understand whether people in the community are willing to be vaccinated against COVID-19 [4], [10].

Since the beginning of the pandemic, Turkish society has been studied to prevent the spread of the disease. From the moment the “COVID-19 (SARS-CoV-2) Infection Guide” was published by the Ministry of Health, it was delivered to all segments of society via written, visual and social media tools in the form of flyers, posters, videos, booklets, and social media about issues such as disease symptoms and ways of disease prevention. Awareness-raising activities have been carried out. In the COVID-19 pandemic, basic public health measures have been carried out as disease control measures under the direction of the political authorities. On the other hand, economic difficulties have also affected decision-makers in determining the framework of the measures. Although the pandemic affects everyone, those who are socioeconomically disadvantaged suffer the most. For many people, it is not possible to work from home, as people with low education and low socioeconomic status are more likely to work in precarious jobs and in the service sector. Poor and deprived populations are less likely to comply with physical distance rules [11]. In these respects, the acceptance of the vaccine is vital in our country. The aim of this study was to determine the acceptance and hesitation regarding vaccination against COVID-19 among Turkish adults.

## Methodology

This descriptive study was conducted in Izmir. The data were collected between October 2020 and January 2021 from a convenience sample of participants. 2023 people who completely filled out the forms were evaluated. The questionnaire, consisting of fifteen items, was delivered via social media and filled in via Google forms. Before starting the survey, participants gave their informed consent and were anonymized. The questionnaire items included socio-demographic characteristics, willingness or hesitation to be vaccinated against COVID-19 and the reasons.

The data were evaluated with the SPSS 22.0. The relationship between the decision to be vaccinated and some characteristics of the individuals was evaluated with the chi-square test. Statistical significance was set at p<0.05.

Permission to conduct the study was obtained from the Ethics Committee of the Ministry of Health and the University of Health Sciences (Number: 2020/11-65).

## Results

The socio-demographic characteristics of the participants are shown in Table 1 (Tab. 1). The mean age of the individuals was calculated as 39.22±15.04 (18-87). Most of the participants were female, in the young age group, employed, living in the province (rural residents), and were university graduates. The mean number of people living at home was calculated as 3.09±1.32 [1], [2], [3], [4], [5], [6], [7], [8], [9], [10], [11], [12]. Self-reported chronic disease frequency was found to be 31.9%. 36.9% of those surveyed smoked. While the majority of the participants had not been vaccinated against pneumonia, there was a higher rate of tetanus vaccinations. As a source of information about COVID-19, respondents mentioned health personnel (24.1%), social media/websites (60.1%), newspapers (12.7%) and television (55.7%).

Of the participants, 68.7% stated that they were willing to be vaccinated against COVID-19 (Table 2 (Tab. 2)). Factors related to the willingness of individuals be vaccinated against COVID-19 are given in Table 3 (Tab. 3) and Table 4 (Tab. 4). The desire for vaccination against COVID-19 was found to be statistically significantly higher among the 50–59 age group, healthcare professionals, non-smokers, living in the city, those with chronic diseases, and those who had been vaccinated against influenza, pneumonia, and tetanus vaccine. Variables such as educational and gender were not found to be associated with the willingness or hesitation to be vaccinated. Those who hesitated to be vaccinated gave various reasons for their hesitation: concern that the vaccine may have side effects, lack of trust in the vaccine because it is a new, lack of belief that the vaccine would work, trust in their own immune system, felt no need to be vaccinated because they are protected from the disease, not afraid of getting sick.

## Discussion

Despite all the known benefits of vaccines, immunization has been challenged throughout history. As a whole, Turkey does not doubt the safety and efficacy of vaccines; however, this study demonstrated negative attitudes towards vaccination and reasons for vaccination hesitancy/rejection such as distrust in the vaccine manufacturers. Especially with the spread of the internet, there has been a serious decrease in the number of parents who have their children vaccinated against common pathogens. A study evaluating the views of adults about general vaccines showed that people were influenced by negative information from social media about vaccines and thus did not trust vaccine manufacturers [12]. It is obvious that the most effective solution to the pandemic today is immunization. On the other hand, willingness to be vaccinated against Covid-19 may be negatively affected by doubts and concerns in society about the safety and suitability of vaccines. Governments need to be prepared to ensure the access and distribution of the COVID-19 vaccine on an equal basis, as well as establish strategies to increase confidence in and acceptance of the vaccine and those who administer it. In order to achieve this, it is important to determine the intention of the society to be vaccinated against COVID-19 and the factors influencing attitudes and behaviors.

In this study, the intention among those surveyed to be vaccinated against COVID-19 was determined as 68.7%. Previously, in a study from May 2020 in Turkey, it was 69% [13]. Our study was carried out during the period when vaccines were available recently and phase studies in Turkey were continuing. In studies conducted in different countries, the acceptance of COVID-19 vaccination varies: 85.8% in Australia [2], 71.5% in a study conducted in 19 countries [14], 73.9% in European countries [1], 86% in England [13], 68.2% in Canada [6], 69% in the USA [15], and United Arab Emirates 75% [16] reported that they were willing to be vaccinated against COVID-19.

In this study, willingness to be vaccinated against COVID-19 was found to be statistically significantly higher among the 50-59 age group, healthcare professionals, non-smokers, urban residents, people with chronic diseases, and those who had been vaccinated against influenza, pneumonia, and tetanus. In previous studies, it was stated that older people, men, more educated people, and those with higher incomes were more willing [2], [13], [14], [16]. In another study, personal characteristics, general vaccination beliefs, knowledge and attitudes about COVID-19 disease and vaccine were determined in models that explained 76% of the intention to be vaccinated against COVID-19 [5]. Unfortunately, gender, social, and economic inequalities affect vulnerability to COVID-19. This situation should not be ignored in providing vaccination services. Inequalities on accessibility of vaccination services and health care in previous SARS (severe acute respiratory syndrome) and Ebola virus outbreaks were deeply understood [17]. It appears that socio-demographic and clinical disparities will become more pronounced if the vaccine is less accepted by certain groups.

The most important reasons for vaccination hesitation in our study were distrust of the vaccine and concerns about its side effects. Similar findings are reported in the literature [1], [2], [18]. Most of these concerns may arise from people’s inability to access accurate and trustworthy information. During the pandemic, combating infodemic (rapid and far-reaching spread of both accurate and inaccurate information about something) has been a major problem. Mis- and disinformation has been spread not only about disease and its treatment, but also about prevention and vaccines. Social media in particular have broadly disseminated such disinformation and made it easily accessible to a very large number of people. The participants in our study belong to the group who use social media the most. In this respect, they may have been impressed by what they “learned” about the vaccine from social media. Public health messages and communication play an important role in informing about vaccination acceptance as well as how to manage risks and prevent transmission during the pandemic.

The limitations of this study are that the data was collected via social media and it could not represent the whole society due to the convenience sample of participants. On the other hand, the results of this study can be used to make recommendations and provide guidance.

## Conclusions

The intention to be vaccinated against COVID-19 was 68.7% in this study. Among the participants, the 50–59 age group, healthcare professionals, non-smokers, urban residents, those with chronic diseases, and those who had previously been immunized against influenza, pneumonia and tetanus were found to be more willing to be vaccinated against COVID-19. It is very important to determine the vaccination willingness of different groups of individuals so that interventions can be made to solve these problems.

## Notes

### Competing interests

The authors declare that they have no competing interests.

### Authors’ contributions participation

All authors participated in the study design, data collection, annotation, manuscript preparation and final approval of the manuscript.

### Data availability

All data are available upon request from the corresponding authors.

### Acknowledgment

This study was conducted with support from Izmir University and TUMS (number 67235) and with their ethical approval (number IR.TBZMED.REC.1399.1071). We thank all participants, volunteer collaborators, and staff of the Department of Infectious Disease for their help during the Covid-19 pandemic.

### ORCID

These are the authors' ORCID IDs:

- Sükran Köse: 0000-0002-4228-1213
- Aliye Mandiracioglu: 0000-0002-0873-4805
- Yusuf Ozbel: 0000-0001-8335-1997
- Seheray Zeyrek: 0000-0003-1579-7588
- Didem Dereli Akdeniz: 0000-0002-5363-6921
- Hossein Samadi Kafil: 0000-0001-6026-8795

## Figures and Tables

**Table 1:**
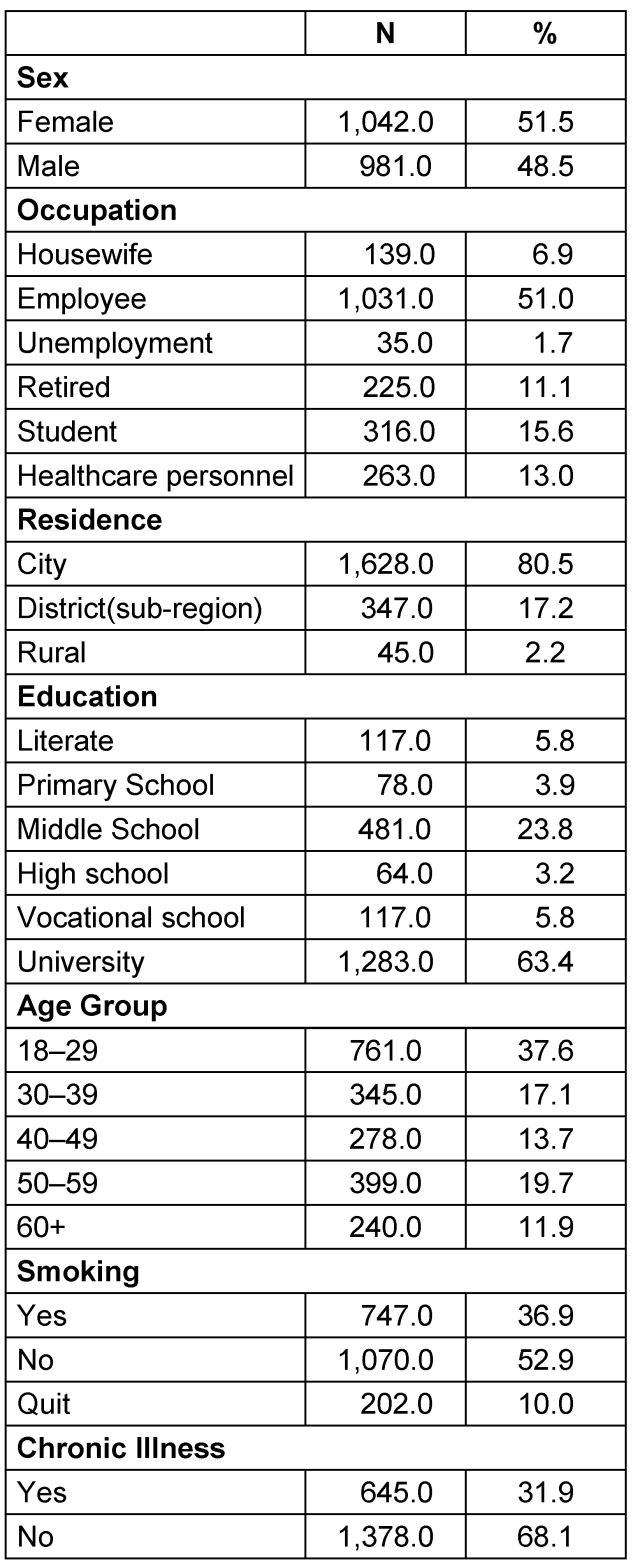
Participant characteristics

**Table 2:**
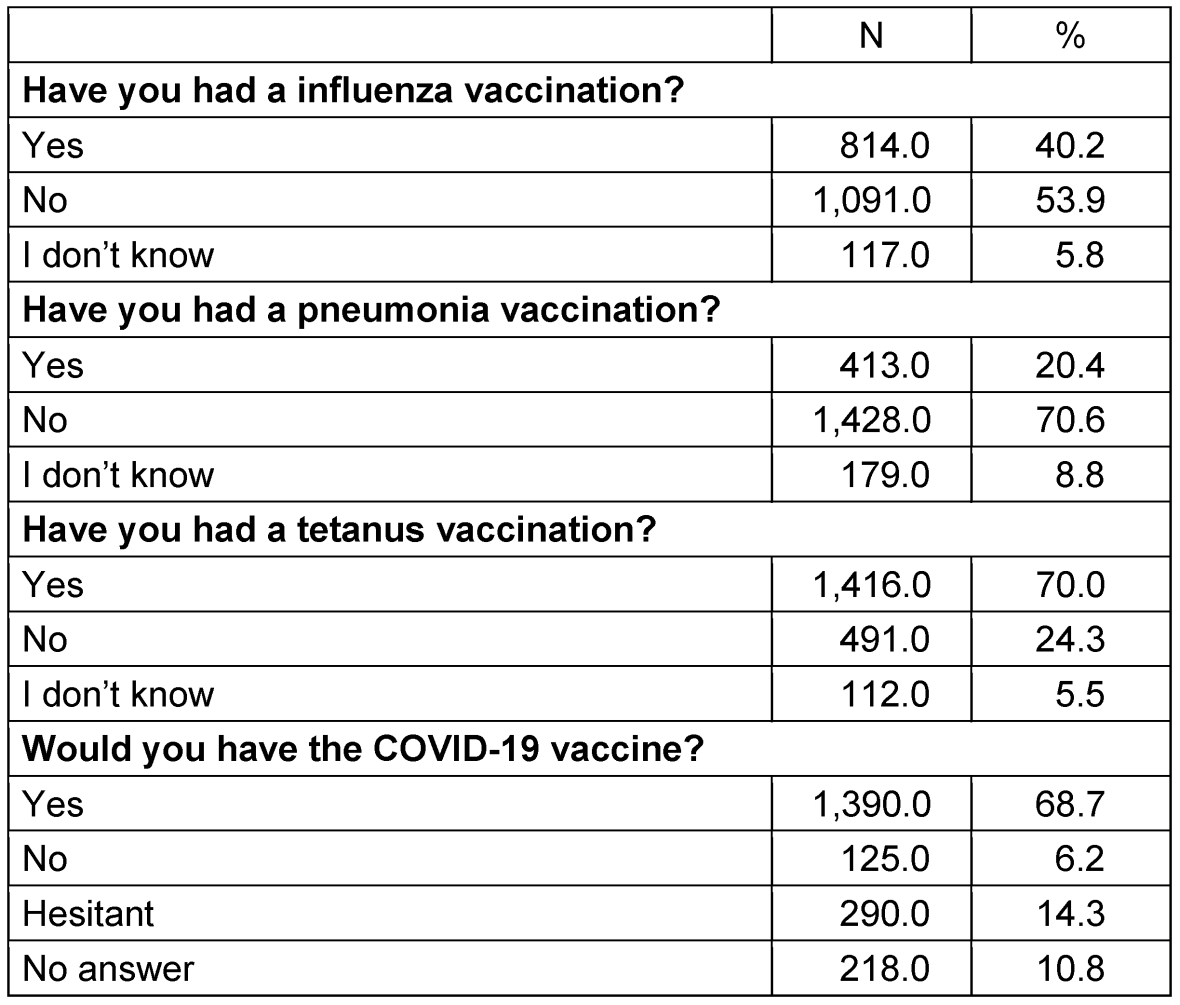
Participants’ prior vaccination status and desire to be vaccinated against COVID-19

**Table 3:**
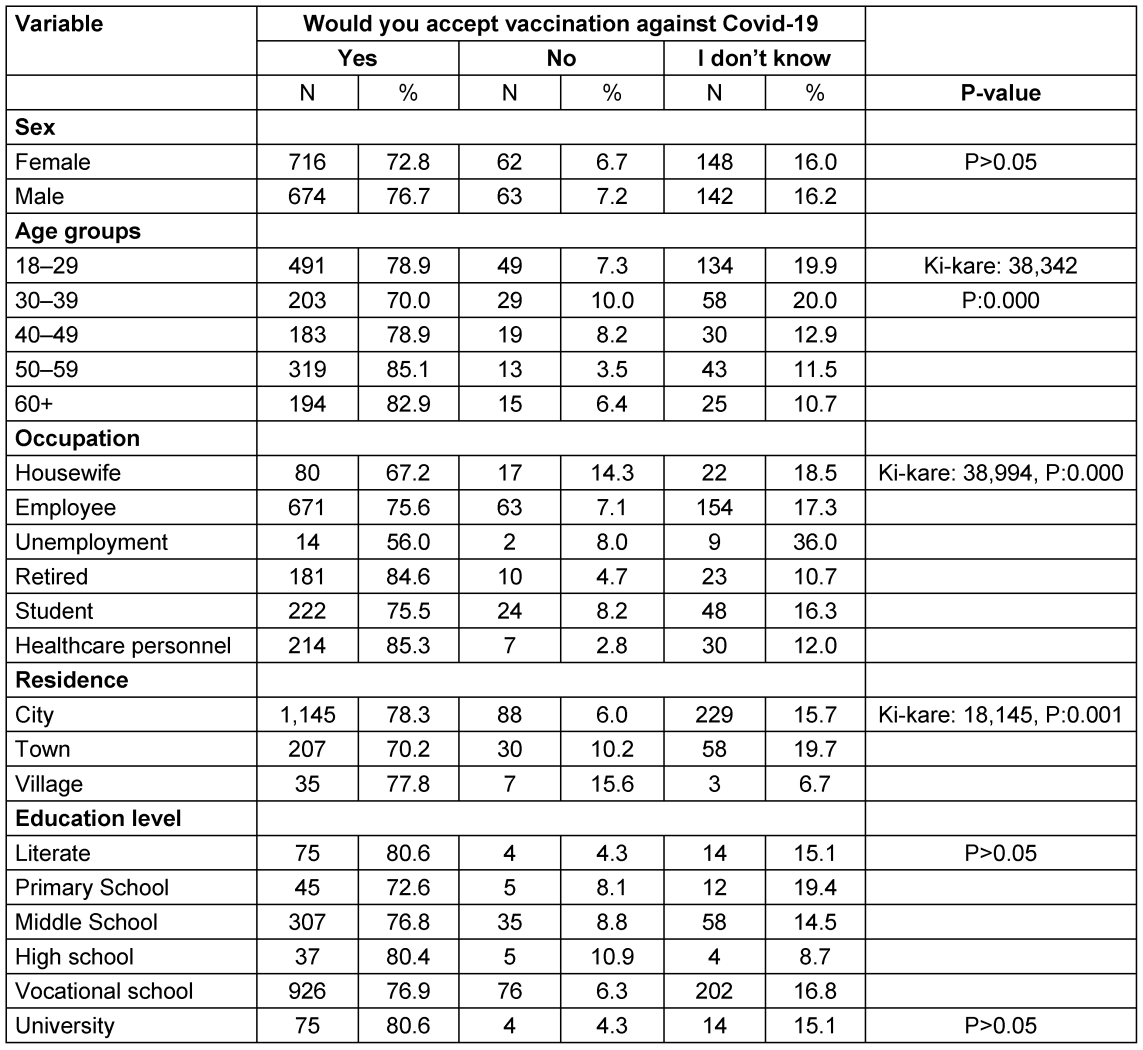
Distribution of factors related to vaccination acceptance

**Table 4:**
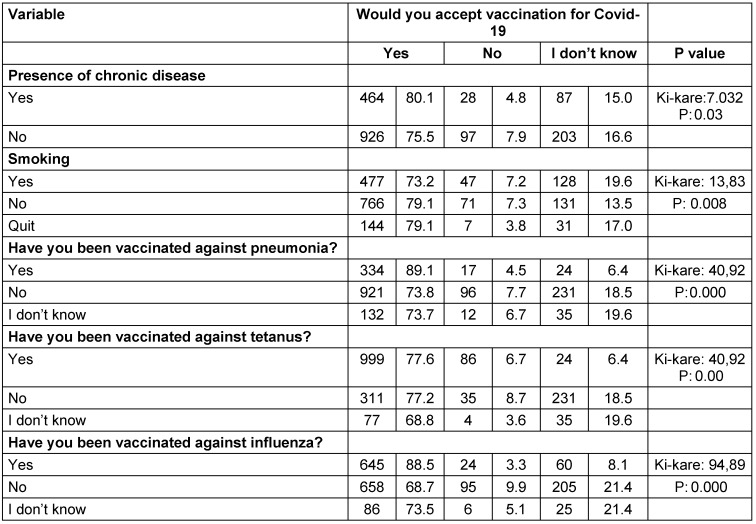
Distribution of factors related to vaccination acceptance
